# Mitochondrial DNA content and oxidation in bipolar disorder and its role across brain regions

**DOI:** 10.1038/s41537-019-0089-5

**Published:** 2019-12-04

**Authors:** D. F. Bodenstein, H. K. Kim, N. C. Brown, B. Navaid, L. T. Young, A. C. Andreazza

**Affiliations:** 1 Department of Pharmacology and Toxicology, University of Toronto, Toronto, ON Canada; 2 Department of Psychiatry, University of Toronto, Toronto, ON Canada; 3 Centre for Addiction and Mental Health, Toronto, ON Canada

**Keywords:** Psychosis, Biomarkers

## Abstract

The underlying pathology of bipolar disorder remains unknown, though evidence is accumulating to support a role of mitochondrial dysfunction. In this study, we aim to investigate electron transport chain complex I subunit NDUFS7 protein expression; mtDNA content; common deletion; and oxidation in the Broadmann area 24 (BA24), cerebellum, hippocampus, and prefrontal cortex from patients with bipolar disorder, schizophrenia, and non-psychiatric controls. Here, we demonstrate no changes in NDUFS7 in BA24, cerebellum or hippocampus, increases in mtDNA content in hippocampus of patients with bipolar disorder, and decreases in mtDNA oxidation in patients with bipolar disorder and schizophrenia, respectively. Paired analysis between BA24 and cerebellum reveal increases within NDUFS7 levels and mtDNA content in cerebellum of patients with bipolar disorder or schizophrenia. We found a positive correlation between NDUFS7 and mtDNA content (ND4 and ND5) when combining brain regions. Our study supports the involvement of mitochondrial dysfunction in bipolar disorder and schizophrenia.

## Introduction

Bipolar disorder (BD) is a severe disorder characterized by alternating episodes of mania and depression, which significantly impact the quality of life and lifestyle of the patient, as well as that of their partners and family members.^1–3^ BD affects roughly 1–3% of the population worldwide,^4^ regardless of socioeconomic status, race or nationality. It is among the leading causes of disability in young adults due to its impact on cognitive and functional capabilities.^1,2^ Patients often report impairment in work, social and familial relations, even after symptoms subside,^2,5^ and are two to three times more likely to divorce or separate from their partners.^3^ Furthermore, patients with BD have ~20 times higher risk of suicide compared to the general population.^1^ Unfortunately, these stressors and challenges can precipitate depressive or manic episodes, leading to further impairment and thus continuing the cycle.^5,6^

There are several hypotheses regarding the pathology of BD, such as the dysregulation of neurotransmitters^5,7^ and intracellular calcium levels,^8^ progressive loss of neurons,^8^ inflammation,^5^ mitochondrial dysfunction, and oxidative stress.^5,9,10^ A number of the above pathways are mediated by the mitochondria. For example, dopamine in the presence of iron spontaneously forms 6-hydroxydopamine, inhibiting complex I of the electron transport chain (ETC).^11–13^ Intracellular calcium levels are regulated by the mitochondria.^14,15^ The nod-like receptor pyrin containing 3 (NLRP3) migrates to the mitochondria forming the NLRP3-inflammasome and activating inflammation following the release of mitochondrial reactive oxygen species (ROS).^16,17^

There is increasing evidence that implicates the mitochondria in BD. Previous work found that patients with BD have significantly decreased activity in complex I of the ETC, specifically in the prefrontal cortex (PFC),^17^ which correlates to the protein level of NDUFS7, a complex I subunit.^10^ Furthermore, microarray studies have found that expression of genes encoding subunits in complex I–V are significantly decreased in BD in the PFC^18^ and hippocampus (HYP).^19^ This decrease in ETC complex activity negatively correlates to the degree of protein carbonylation and nitration.^10^ Furthermore, mitochondrial DNA (mtDNA) is particularly susceptible to oxidative damage, due to a lack of protective histones,^20^ which can result in mutations and large deletions with most occurring within the major arc.^21^

One of the most common causes of mitochondrial disease is mtDNA depletion.^22^ Early discoveries from Kato et al.^23^ found significant increases in mtDNA deletion in post-mortem brain tissue of patients with BD, thus supporting the theory of mtDNA deletion and depletion in the pathology of BD.^23^ However, despite this early evidence, multiple studies have since found mtDNA content and deletions to be unchanged.^20,24–26^ Mamdani et al.^26^ found no change in mtDNA deletion in patients with BD across multiple brain regions. Other studies within the occipital and frontal cortex have revealed similar findings, where no change was found in mtDNA deletion in patients with BD.^20,24,27^ However, this is contradicted by Sequeira et al. and Shao et al. that found significant increases in mtDNA deletion in BD compared to non-psychiatric controls in the dorsolateral prefrontal cortex (DLPFC).^28,29^ To the best of our knowledge, there are no studies investigating mtDNA oxidation in post-mortem brain tissue of patients with BD.

Taken all together, there is an apparent need to investigate mtDNA content and damage across brain regions in patients with BD and schizophrenia (SCZ). In this study, we investigated NDUFS7 protein expression, and mtDNA content, deletion and oxidation across four brain regions, specifically the PFC, HYP, Broadmann area 24 (BA24), and cerebellum (CE) in BD, SCZ, and non-psychiatric controls (CTL). We have chosen the above brain regions based on previous reports, which have shown their association with the pathology of BD and SCZ, mitochondrial dysfunction and oxidative stress, and based on tissue availability from biobanks. Based on previous findings from our laboratory and other studies, we expect to find decreases in mtDNA content and increases in mtDNA oxidation and common deletion in the PFC.

As previously published, ETC complex I subunit NDUFS7 was decreased in the PFC of patients with BD compared to CTL.^17^ Here, we demonstrated no changes in NDUFS7 in other brain regions, increased levels of mtDNA content in HYP of patients with BD and decreased mtDNA oxidation in BA24 and CE of patients with BD and SCZ, respectively. We investigated region specific effect among same patients. Paired analysis between BA24 and CE revealed increased NDUFS7 levels and mtDNA content in CE of patients with BD or SCZ but not in CTL. Also, we evaluated the relationship between NDUFS7 levels and mtDNA content of complex I subunits. The results show a positive correlation between NDUFS7 and mtDNA content of complex I subunits when brain regions are combined. Lastly, we demonstrate no alteration in the levels of mtDNA deletion between different diagnoses. Altogether, the results suggest that different brain regions have unique susceptibility to change in the energy metabolism, warranting further investigation.

## Results

### Post-mortem brain samples demographics

Information regarding demographics of individuals from whom brain tissue was collected can be found in Table 1. Tissue samples from the BA24 and CE were obtained from the same patients. Age and PMI did not differ significantly between diagnosis groups in HYP and PFC samples. However, in BA24 and CE the PMI was significantly reduced in BD patients. Age did not differ in BA24 and CE patients.

**Table 1:** Sample demographics for all samples within each brain region

Brain region		CTL	BD	SCZ	*p*-value
Hippocampus		*N* = 19	*N* = 12	*N* = 15	
	Age (years)	59.5 ± 16.1	58.5 ± 23.6	62.4 ± 14.2	0.832
	PMI (hours)	22.2 ± 3.4	20.7 ± 4.5	22.7 ± 7	0.591
Broadmann area 24 + cerebellum^a^		*N* = 10	*N* = 10	*N* = 10	
	Age (years)	71 ± 8.4	72 ± 5.6	68.9 ± 13.1	0.763
	PMI (hours)	23.8 ± 4.3	16.7 ± 7.1	25.8 ± 5.7	0.004^a^
Prefrontal cortex^b^		*N* = 8	*N* = 9	*N* = 10	
	Age (years)	71.6 ± 15.6	64.83 ± 15.1	71.3 ± 12.4	0.631
	PMI (hours)	19.1 ± 2.9	21.8 ± 7.7	20.9 ± 9.9	0.815

PMI is significantly decreased in BD compared to CTL and SCZ patients in the BA24 and CE through ANOVA

Broadmann area 24 (BA24) and cerebellum (CE) tissue samples are collected from the same patients

*PMI* post-mortem interval

^a^Note that tissue samples for cerebellum were available from CTL = 8, BD = 10, SCZ = 9, while CTL = 10, BD = 10, SCZ = 10 tissue samples were available for BA24, thus the demographics reflect ten individuals per group

^b^Demographic characteristics of PFC samples reflect samples used in the mtDNA content, deletion and oxidation analysis. Demographics characteristics from PFC samples used for NDUFS7 levels can be found at Kim et al.^17^

### Mitochondrial complex I subunit NDUFS7 levels

**Fig. 1:**
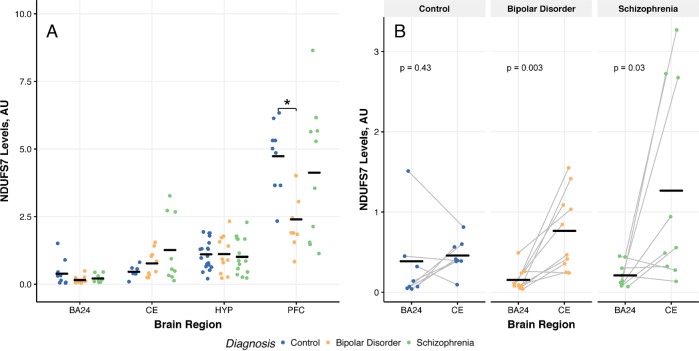
NDUFS7 levels across brain regions from patients with bipolar disorder or schizophrenia. **a** Levels of complex I subunit NDUFS7 across the brain regions. NDUFS7 levels were normalized to β-actin, an established loading control. Significance was measured through non-parametric Kruskal–Wallis test with Dunn test post-hoc with Bonferroni correction. See Supplementary Fig. 1 for representative western blots. **b** Comparison of NDUFS7 protein levels between BA24 and CE since tissue was obtained from the same patients. Significance was tested via paired *t*-test. *p < 0.05, Kruskal–Wallis compared to control

As previously published by Kim et al., NDUFS7 protein levels were reduced in the PFC of patients with BD compared to CTL through non-parametric Kruskal–Wallis followed by Dunn test post-hoc with Bonferroni correction (Fig. 1a).^17^ We found no changes in the protein levels of NDUFS7 in the BA24, HYP, and CE (Fig. 1a). We performed paired comparison via *t*-test to assess brain region on NDUFS7 protein levels in samples collected from the BA24 and CE, since samples were obtained from the same individuals. Here, we demonstrated increases in NDUFS7 protein level within the CE compared to BA24 of patients with BD and SCZ, but not in CTL (Fig. 1b).

### mtDNA content

**Fig. 2:**
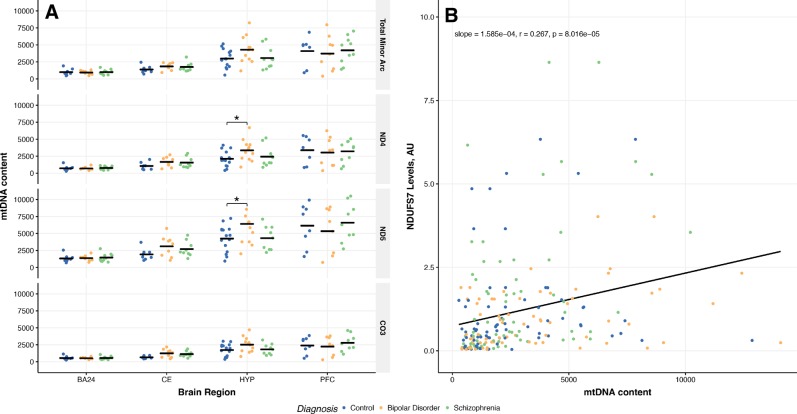
mtDNA content across brain regions from patients with bipolar disorder or schizophrenia. **a** mtDNA content as measured by qPCR, normalized to β2-microglobulin in the nuclear genome. Total minor arc is the mean of the MT-tRNA and MT-ND1 to provide total mtDNA content of the minor arc. Major arc genes, MT-ND4, MT-ND5, and MT-CO3, were not combined as they reside in the major arc, which is prone to deletions. Significance was measured through one-way followed by Tukey HSD post-hoc, in addition to ANCOVA to control for age and PMI. **b** Correlation of NDUFS7 levels and MT-ND4 and MT-ND5 content from samples obtained from all brain regions of patients with BD, SCZ, and non-psychiatric controls. Results were analyzed using Pearson correlation test. See Supplementary Tables 2 and 3 for complete information regarding panels **a** and **b**. *p < 0.05, ANCOVA with PMI compared to CTL

The results reveal that in HYP samples, mtDNA content for MT-ND4 and MT-ND5 were significantly increased in patients with BD compared to CTL, controlling for PMI as it demonstrated a correlation with mitochondrial DNA content (Fig. 2a). We also investigated the brain region effect of mtDNA content among BA24 and CE, demonstrating higher levels of mtDNA content in CE of patients with BD or SCZ, but not CTL (Supplementary Fig. 1). We also investigated the relationship between NDUFS7 and mtDNA content of complex I subunits. Our results show a positive correlation between NDUFS7 and mtDNA content of complex I subunits (Fig. 2b). This relationship demonstrated a stronger correlation for patients with SCZ, followed by patients with BD and not significant for CTL (Supplementary Table 3).

### mtDNA deletion

**Fig. 3:**
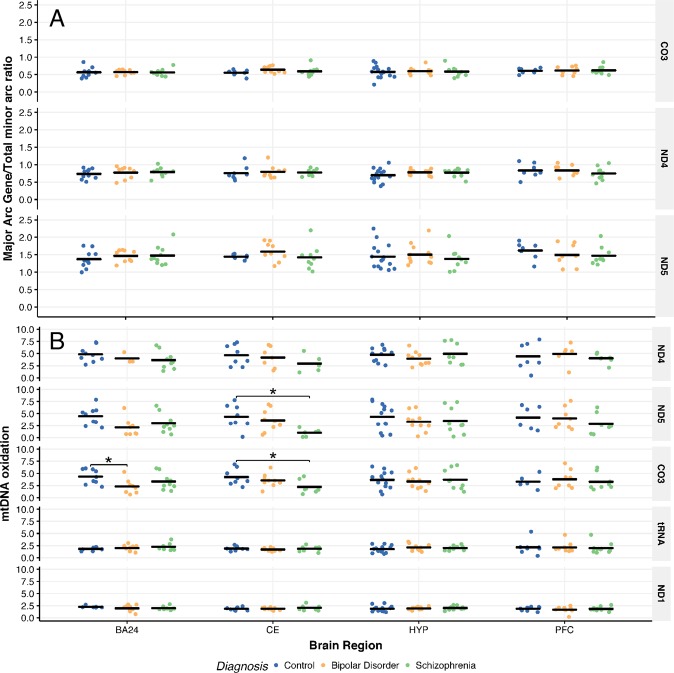
mtDNA damage across brain regions from patients with bipolar disorder or schizophrenia. **a** Estimate of the common deletion provided by the ratio of the mtDNA content of major arc genes, MT-ND4, MT-ND5, and MT-CO3, to the total minor arc mtDNA content. Significance was measured through 1-way ANOVA followed by Tukey HSD post-hoc, in addition to ANCOVA to control for age and PMI. **b** mtDNA oxidation as measured by qPCR following treatment with or without FPG, expressed as ΔC_t_. Significance was measured through one-way ANOVA followed by Tukey HSD post-hoc. See Supplementary Tables 4 and 5 for complete results and information on samples sizes and mean for panels **a** and **b**. *p < 0.05, ANOVA compared to CTL

mtDNA deletion was assessed as the ratio of mtDNA content of major arc genes to the total mtDNA content of the minor arc genes, as adapted from the protocol of Kakiuchi et al. and Torrell et al.^20,25^ This protocol was utilized as a way to estimate the extent of the common deletion occurring in the major arc. As the ratio between the major and minor arc should be consistent in the absence of deletions, utilizing the ratio allows us to estimate deletions within the major arc in patients with BD and SCZ compared to CTL. Overall, we found no significant alterations in mtDNA deletion between diagnoses across the brain regions (Fig. 3a). There were no significant between-group differences when age and PMI were controlled for as covariates via analysis of covariance (ANCOVA) as well.

### mtDNA oxidation

In the BA24, oxidative damage in MT-CO3 was significantly reduced in BD compared to CTL (Fig. 3b). Similarly, in the CE, we found significant reductions in oxidative damage in SCZ compared to CTL in MT-CO3 and MT-ND5 in the major arc (Fig. 3b). As shown in Fig. 3b, mtDNA oxidation in the major arc is generally higher (Δ*C*_t_ 1–4.9) when compared to mtDNA oxidation levels in the minor arc (Δ*C*_t_ 1.7–2.2) genes.

## Discussion

Mitochondrial dysfunction plays an important role in BD in the prefrontal cortex demonstrated by reduced ETC complex assembly and increased oxidative damage.^9,10,17^ mtDNA depletion syndrome is the most common cause of reduced ETC complex assembly and is a major source of ROS. Thus, we expected to find decreased NDUFS7 and mtDNA content and an increase in oxidation in the PFC. We demonstrated a brain region-dependent effect (BA24 and CE) on the levels of NDUFS7 expression and mtDNA content that is only present within patients with BD and SCZ, in addition to significantly reduced NDUFS7 levels in the PFC of patients with BD. Unexpectedly, we found significant increases in mtDNA content in the HYP in patients with BD and decreased oxidation in BA24 and CE from patients with BD and SCZ, respectively. Taken together, the results reveal a unique vulnerability of different brain regions to energetic demands and suggests a potential underlying compensatory mechanism within the cells and brain tissue to maintain mitochondrial mass.

Here, we demonstrated increased mtDNA content in the HYP of patients with BD and no changes in other brain regions, as well as no changes in the mtDNA deletions levels. Previous studies have not been conclusive in determining whether mtDNA content and deletions are increased or decreased in post-mortem brain tissue of patients with BD and SCZ, specifically within the frontal and occipital cortex.^20,24–29^ However, it is possible that we, and other groups, may underestimating total mtDNA deletions, since next-generation sequencing was not performed.

In this study, we aimed to investigate the brain region-specific effect among BA24 and CE (from same patients) to understand whether there is a unique susceptibility to changes in mtDNA across regions within the same participant. We found significant brain region dependent effects through paired *t*-test, where the CE had significantly higher mtDNA content compared to BA24 within patients with BD and SCZ, but not within CTL (Supplementary Fig. 2). Interestingly, most findings and studies in BD have been conducted in the PFC. Microarray studies within the PFC and HYP have also found significant reductions in expression of genes within the ETC, such as NDUFS7 and NDUFS8.^18,19,30^ However, a recent finding by Gandal et al.^31^ utilizing RNA-sequencing demonstrated no difference in the expression levels of NDUFS7 and NDUFS8 in cortical tissue of patients with BD,^31^ emphasizing the complexity and heterogeneity of BD. Despite conflicting microarray and RNA-sequencing results, previous work has repeatedly found significantly reduced NDUFS7 protein levels within the PFC of patients with BD, as well as reduced ETC complex I activity.^10,17^ These previous findings alongside our results lead us to believe that complex I subunit NDUFS7 and the PFC play a fundamental role within the pathology of BD and should be continuously studied in order to aid the discovery of the underlying mechanisms and improve treatment regimens for patients with BD.

mtDNA is particularly susceptible to oxidative damage due to the lack of protective histones.^20^ This increased susceptibility can result in increased damage and deletions, with the most common being a 4977 base pair deletion within the mtDNA major arc, called the common deletion.^20^ Interestingly, we found increases in mtDNA oxidation within the major arc compared to the minor arc, indicative of an increase in mtDNA damage within the major arc, which may explain the formation of deletions within this region. Additionally, our results found significant reductions in oxidative lesions in patients with BD and SCZ compared to CTL, specifically within the major arc genes, MT-ND5 and MT-CO3, in the BA24 and CE, respectively. Furthermore, CE was found to have a role in the pathophysiology of SCZ.^32,33^ Altogether these findings potentially indicate compensatory mechanisms preserving the mtDNA integrity within the CE from downstream alterations in SCZ. Alternatively, Kim et al.^34^ demonstrated that nitration levels of tyrosine hydroxylase are decreased and oxidation levels of dopamine transporter are increased in patients with BD and SCZ in the PFC. This disruption within the dopaminergic system leads to increased generation of ROS through the metabolism of dopamine and indirectly through the inhibition of the ETC by dopamine quinones,^35^ as well as opening of the mitochondrial permeability transition pore (mPTP).^11^ mPTP opening is believed to act as mechanism to release generated ROS,^36^ which may direct it away from mtDNA within the mitochondrial matrix, therefore, reducing levels of mtDNA oxidation.

In patients with BD NDUFS7 is decreased in PFC and unchanged in other regions, mtDNA content is increased in HYP, and mtDNA oxidation is decreased in BA24, as well as decreased in patients with SCZ in CE. Brain region-specific changes in mitochondria were investigated in the BA24 and CE among the same patients. Paired analysis between BA24 and CE revealed increased NDUFS7 levels and mtDNA content in CE of patients with BD and SCZ, but not in CTL. This potentially suggests unique susceptibility to alterations in energy metabolism within distinct brain regions that is not present in CTL. Additionally, a positive correlation was found between NDUFS7 and mtDNA content of mitochondrial complex I subunits (ND4 and ND5) when combining brain regions. We found no alteration in the levels of mtDNA deletion between different diagnoses. These findings support a role of mitochondrial dysfunction in the pathology of BD and SCZ, and support the importance of the PFC and CE within each disease, respectively.

This study is subject to the limitations associated with post-mortem tissue. mtDNA content has been shown to decrease after middle age,^37,38^ while mtDNA deletions accumulate with age.^26,27^ Therefore, age is an important covariate to control in analysis of mtDNA. pH of the brain tissue was not available for all brain regions, which was found to be an important covariate in analysis of mtDNA content by Vawter et al.^21^ PMI can be used as an indicator of autolytic degradation, similar to pH.^21,26^ However, PMI and age were available for all brain regions, and were used as covariates in ANCOVA where applicable. It is difficult to control for the effects of medications, such as anti-psychotics and anti-depressants, and patient and family history. There are changes in cellular signaling and mechanisms depending on the state of BD patients—depressive, manic, or euthymic—at the time of tissue collection.^7^ Furthermore, due to the scarcity of post-mortem tissue, the tests that can be performed are often limited to tests that use low quantities of tissue to allow for the most tests to be done. Additionally, aside from the BA24 and CE regions originating from the same patients, the PFC and HYP were obtained from different patients, meaning we cannot measure changes in NDUFS7 expression and mtDNA content and damage across the brain regions within the same patients in all regions.

**Fig. 4:**
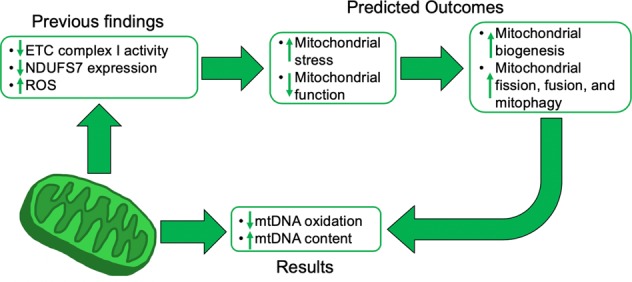
Proposed compensatory mechanism to maintain mitochondrial function and integrity. Increased stress within the cells, due to aberrant mitochondrial function from decreased ETC activity and complex I subunit, NDUFS7, mRNA and protein expression, potentially activate mitochondrial biogenesis to increase mitochondrial function. Additionally, mitochondrial dysfunction increases the production of mitochondrial ROS, activating mitochondrial fission, fusion, and mitophagy pathways to remove damaged mtDNA, as well as activating mitochondrial biogenesis. The combination of these pathways may explain the decrease in mtDNA oxidation and increase in mtDNA content that we observed in patients with BD and SCZ

Taken together, we believe these results represent a compensatory mechanism to maintain mitochondrial function and DNA integrity via the upregulation of mitochondrial biogenesis and stress response pathways (i.e. metabolites oxidation or fusion/fission; Fig. 4). Mitochondrial biogenesis may be increased to compensate for significantly reduced mRNA and protein expression of ETC complex I subunit, NDUFS7,^10,17–19^ which has been shown to be closely linked with mitochondrial function.^10^ Furthermore, these reductions in NDUFS7 expression and associated mitochondrial dysfunction would cause increased oxidative stress,^10^ activating both mitochondrial biogenesis and stress response pathways, specifically fusion, fission, and mitophagy.^39–41^ This would lead to the removal of damaged mitochondria and DNA, effectively reducing the level of measured oxidatively damaged mtDNA. Therefore, we believe the compensatory effects of increased mitochondrial biogenesis and stress response pathways can explain the results that demonstrated significantly increased mtDNA content and significantly decreased mtDNA damage in patients with BD and SCZ compared to CTLs. Further research is required to explore the role of mitochondrial genetics in psychiatric conditions to aid in a better understanding of the underlying pathology.

## Methods

### Post-mortem brain samples

Tissue samples were obtained from the Harvard Brain Tissue Resources (http://www.brainbank.mclean.org/). Post-mortem brain samples were separated by diagnosis: BD, SCZ, and CTL, see Table 1 for complete details. Tissue was further separated by brain region: (1) BA24 (BD-n = 10, SCZ-n = 10, CTL-n = 10), (2) CE (BD-n = 10, SCZ-n = 9, CTL-n = 8), (3) HYP (BD-n = 12, SCZ-n = 15, CTL-n = 19), and (4) PFC (BD-n = 9, SCZ-n = 10, CTL-n = 8). Samples received from BA24 and CE are from the same individuals, whereas samples from the HYP and PFC are from different individuals. Therefore, only BA24 and CE were used for analysis of brain region specificity. Throughout all experiments, samples were assigned random numerical identities to ensure researchers were blind to the brain region and diagnosis of each sample. The code was only lifted upon completion of the experiment to allow for analysis of results. Age and post-mortem interval (PMI) information was provided with the tissue for use as covariates. Sample demographics and information can be found in results and Table 1.

### Mitochondrial complex I subunit NDUFS7 levels

Mitochondrial complex I subunit NDUFS7 protein levels were measured using immunoblotting as described by Kim et al.^17^ Whole-tissue homogenates were diluted to a final concentration of 10 µg tissue/15 µl sodium dodecyl sulfate–polyacrylamide gel electrophoresis (SDS-PAGE) sample buffer (50 mM Tris HCl (pH 6.8), 100 mM DTT, 2% SDS, 0.1% bromophenol blue, 10% glycerol). Samples were then loaded onto a 12% acrylamide sodium dodecyl sulfate–polyacrylamide electrophoresis gel, which was then transferred to a polyvinylidene difluoride (PVDF) membrane. The membrane was then incubated for 2 h at room temperature with the primary antibody (NDUFS7 [Santa Cruz Biotechnology, #98644]), followed by 1-h incubation at room temperature with a horseradish peroxidase conjugated secondary antibody. Bands were detected with ECL reagents and analyzed densitometrically via Versa Doc from Bio-Rad (Bio-Rad Laboratories Ltd., Mississauga, Canada). Blots were normalized against β-actin via an anti-β-actin antibody (Abcam Inc, #ab8226), which is an established loading control.

### Mitochondrial DNA content and deletion

The protocol used to measure the mtDNA content was modified from a previously published method from Venegas & Halberg.^22^ mtDNA content was measured by quantitative PCR (qPCR) using 6 primer pairs: (1) MT-tRNA (mtDNA), (2) MT-ND1 (mtDNA), (3) MT-ND4 (mtDNA), (4) MT-ND5 (mtDNA), (5) MT-CO3 (mtDNA), and (6) β2-microglobulin (nDNA) (Table 2). Primers were validated using a geneblock (Integrated DNA Technologies, Coralville, Iowa, Table 2) and a reference sample created by combining 5 μl of extracted DNA from each of the brain tissue samples to better represent experimental conditions. Following testing, specificity was confirmed by analyzing the melt-curve of each primer. β2-microglobulin was used to control for variations in the starting DNA quantity, as it is a one-copy gene within the nuclear genome. DNA was extracted from the brain samples using the Qiagen QIAmp DNA Mini kit (Catalog No. #51306). Extracted DNA was then diluted to 0.1 ng/µl and plated with Bioline 2x SensiFAST SYBR No-ROX (Catalog No. #BIO-98050). The qPCR was performed with the Bio-Rad CFX96 (Bio-Rad Laboratories, Inc.) with the following cycling conditions: (1) 95 °C for 3 min; (2) 95 °C for 10 s and; (3) 60 °C for 20 s for 40 cycles. Following completion of qPCR, the ∆*C*_t_ was calculated for each sample (∆*C*_t_ = (average mtDNA C_t_) – (average nDNA C_t_)), from which the mtDNA content was calculated (mtDNA content = 2^−∆Ct^). The mtDNA content for MT-ND1 and MT-tRNA were combined using their mean to calculate the total minor arc content. In order to estimate the extent of mtDNA deletion, ratio was obtained using the mtDNA content of the major arc genes to the total minor arc content and compared across diagnosis groups, as adopted from the protocol of Kakiuchi et al. and Torrell et al.^20,25^

**Table 2:** Primer sequences used for qPCR to assess mtDNA content and damage, and geneblock

Gene	Forward primer	Reverse primer	Geneblock
ß2-microglobulin	5′-TGCTGTCTCCATGTTTGATGTATCT-3′	5′-TCTCTGCTCCCCACCTCTAAGT-3′	5′-TGCTGTCTCCATGTTTGATGTATCTGAGCAGGTTGCTCCACAGGTAGCTCTAGGAGGGCTGGCAACTTAGAGGTGGGGAGCAGAGA-3′
MT-tRNA	5′-CACCCAAGAACAGGGTTTGT-3′	5′-TGGCCATGGGTATGTTGTTA-3′	5′-CACCCAAGAACAGGGTTTGTTAAGATGGCAGAGCCCGGTAATCGCATAAAACTTAAAACTTTACAGTCAGAGGTTCAATTCCTCTTCTTAACAACATACCCATGGCCA-3′
MT-ND1	5′-ATGGCCAACCTCCTACTCCT-3′	5′-CTACAACGTTGGGGCCTTT-3′	5′-CACCCAAGAACAGGGTTTGTTAAGATGGCAGAGCCCGGTAATCGCATAAAACTTAAAACTTTACAGTCAGAGGTTCAATTCCTCTTCTTAACAACATACCCATGGCCA-3′
MT-ND4	5′-ACAATCTGATGTTTTGGTTAAACTATATTT-3′	5′-CCATTCTCCTCCTATCCCTCAAC-3′	5′-CCATTCTCCTCCTATCCCTCAACCCCGACATCATTACCGGGTTTTCCTCTTGTAAATATAGTTTAACCAAAACATCAGATTGT-3′
MT-ND5	5′-ATAACCATGCACACTACTATAACCA-3′	5′-GTTAACGAGGGTGGTAAGGATG-3′	5′-ATAACCATGCACACTACTATAACCACCCTAACCCTGACTTCCCTAATTCCCCCCATCCTTACCACCCTCGTTAAC-3′
MT-CO3	5′-CGAGTCTCCCTTCACCATTTC-3′	5′-TTGGCGGATGAAGCAGATAG-3′	5′-CGAGTCTCCCTTCACCATTTCCGACGGCATCTACGGCTCAACATTTTTTGTAGCCACAGGCTTCCACGGACTTCACGTCATTATTGGCTCAACTTTCCTCACTATCTGCTTCATCCGCCAA-3′

### Mitochondrial DNA oxidation

The protocol used to measure the mtDNA oxidation was modified from a previously published method from Lin et al.^42^ mtDNA oxidation was measured by quantitative PCR (qPCR) using five primer pairs: (1) MT-tRNA (mtDNA), (2) MT-ND1 (mtDNA), (3) MT-ND4 (mtDNA), (4) MT-ND5 (mtDNA), and (5) MT-CO3 (mtDNA) (Table 2). Primers were validated using a geneblock (Integrated DNA Technologies, Coralville, Iowa, Table 2) and a reference sample created by combining 5 μl of extracted DNA from each of the brain tissue samples to better represent experimental conditions. Following testing, specificity was confirmed by analyzing the melt-curve of each primer. DNA was extracted from the brain samples using the Qiagen QIAmp DNA Mini kit (Catalog No. #51306). Extracted DNA was then diluted to 1 ng/µl and treated with New England BioLabs formamidopyrimidine DNA glycosylase (FPG; Catalog No. #M0240L) or with an equivalent volume of H_2_O. Treatment mixtures were then incubated for 1-h at 37 °C, followed by a 10-min inactivation step at 60 °C, and stored at 4 °C until used. Upon use, treated DNA is combined with Bioline 2x SensiFAST SYBR No-ROX (Catalog No. #BIO-98050). The qPCR was performed with the Bio-Rad CFX96 (Bio-Rad Laboratories, Inc.) with the following cycling conditions: (1) 95 °C for 3 min; (2) 95 °C for 10 s and; (3) 60 °C for 20 s for 40 cycles. Following completion of qPCR, the ∆C_t_ was calculated for each sample (∆C_t_ = (average C_t_ of FPG treated) – (average C_t_ of FPG untreated)).

### Statistics methods

Statistics were performed using R version 3.5.2,^43^ with packages dplyr^44^ and ggplot2.^45^ Prior to any statistical analysis, results were separated into each brain region and tested for normality using a Shapiro–Wilks test. If it failed the normality test, non-parametric analysis through Kruskal–Wallis test was performed, and any outliers (defined as any value less than or greater than the first or third quartile by >1.5 times the interquartile range) were removed and re-tested for normality. If removing outliers resulted in passing the normality test, one-way ANOVA was performed controlling for diagnosis followed by a Tukey HSD post-hoc. Analysis was performed gene by gene within each brain region in order to avoid multiple comparisons, therefore limiting them only to diagnosis (CTL to BD, CTL to SCZ, BD to SCZ). It was a conservative approach to avoid the effect of sample size, power, and unreliable data. Pearson’s correlation was measured with each covariate (age and post-mortem interval [PMI]), which if significant was followed by an ANCOVA. Relationship between NDUFS7 and mtDNA content between brain regions were assessed through paired *t*-test and Kruskal–Wallis. Similarly, the relationship between oxidative damage and mtDNA within the minor arc compared to the major arc was determined through a *t*-test.

## Supplementary information

Supplemental Figures and Tables

## Data Availability

The datasets generated during and/or analyzed during the current study are available from the corresponding author on reasonable request.
