# Supplementary material for: Mitochondrial DNA content and oxidation in bipolar disorder and its role across brain regions
Source: NPJ Schizophr. 2019 Dec 4;5:21. doi: 10.1038/s41537-019-0089-5 (PMC6892804; doi:10.1038/s41537-019-0089-5)
Supplement: Supplementary file 1 — Supplemental Figures and Tables [file 41537_2019_89_MOESM1_ESM.pdf]

Supplementary Tables

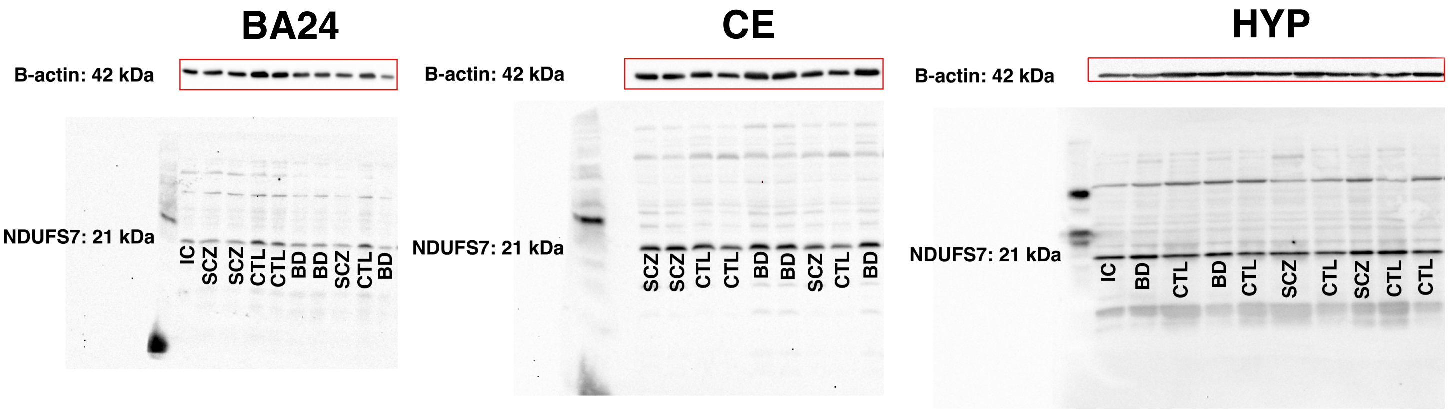

Supplementary Figure 1. Representative western blots used to evaluate complex I subunit NDUFS7 protein levels for BA24, CE, and HYP. A representative western blot for the PFC can be found in Figure 1 of Kim *et al*. (2016)[17].

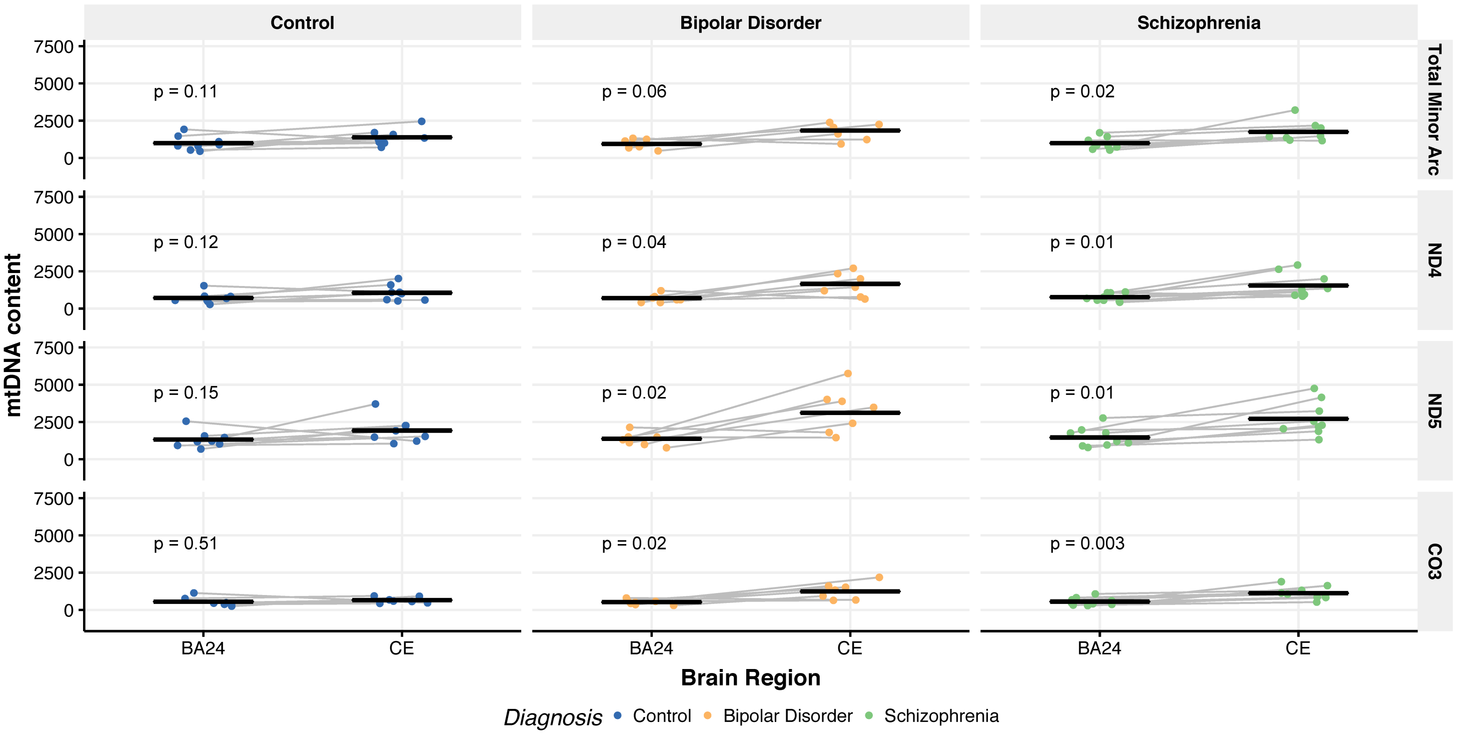

Supplementary Figure 2. Comparison of mtDNA content between BA24 and CE since tissue was obtained from the same patients. Significance was tested via paired t-test.

Brain Region	Diagnosis	N	Mean	SD	SEM
BA24	Control	10	0.386	0.475	0.150
	Bipolar Disorder	10	0.152	0.141	0.044
	Schizophrenia	10	0.210	0.142	0.045
CE	Control	8	0.457	0.209	0.074
	Bipolar Disorder	10	0.766	0.488	0.154
	Schizophrenia	9	1.265	1.249	0.416
HYP	Control	19	1.104	0.535	0.123
	Bipolar Disorder	11	1.119	0.692	0.209
	Schizophrenia	15	1.012	0.655	0.169
PFC	Control	9	4.735	1.293	0.431
	Bipolar Disorder	9	2.397	1.097	0.366
	Schizophrenia	10	4.124	2.528	0.799

Supplementary Table 1. Information on sample size, mean, standard deviation, and standard error of the mean for NDUFS7 protein levels (Figure 1A).

Brain Region	Diagnosis	Gene	N	Mean	SD	SEM
BA24	Control	Total Minor Arc	8	994.736	488.538	172.724
		ND4	8	717.251	377.376	133.422
		ND5	8	1326.248	569.306	201.280
		CO3	8	553.429	279.473	98.809
	Bipolar Disorder	Total Minor Arc	8	939.018	299.063	105.735
		ND4	8	699.089	261.034	92.290
		ND5	8	1373.169	430.321	152.142
		CO3	8	522.886	158.045	55.877
	Schizophrenia	Total Minor Arc	10	991.668	369.788	116.937
		ND4	10	765.169	259.916	82.193
		ND5	10	1457.824	612.561	193.709
		CO3	10	558.018	246.542	77.964
CE	Control	Total Minor Arc	8	1380.733	536.911	189.827
		ND4	8	1059.997	527.386	186.459
		ND5	8	1927.009	846.744	299.369
		CO3	7	655.691	201.009	75.974
	Bipolar Disorder	Total Minor Arc	7	1835.811	584.063	220.755
		ND4	8	1657.356	755.618	267.151
		ND5	9	3115.950	1535.790	511.930
		CO3	9	1239.645	600.087	200.029

	Schizophrenia	Total Minor Arc	8	1748.588	694.119	245.408
		ND4	9	1549.788	781.677	260.559
		ND5	9	2709.138	1122.861	374.287
		CO3	9	1127.555	426.093	142.031
HYP	Control	Total Minor Arc	15	2993.612	1328.241	342.950
		ND4	15	2113.992	1137.242	293.635
		ND5	15	4256.215	1919.578	495.633
		CO3	15	1730.051	895.807	231.296
	Bipolar Disorder	Total Minor Arc	12	4290.267	1951.032	563.214
		ND4	12	3358.171	1572.791	454.026
		ND5	12	6410.795	3160.138	912.253
		CO3	12	2522.540	1120.793	323.545
	Schizophrenia	Total Minor Arc	10	3073.535	1622.296	513.015
		ND4	10	2424.484	1495.596	472.949
		ND5	9	4316.707	1788.102	596.034
		CO3	9	1826.688	786.673	262.224
PFC	Control	Total Minor Arc	7	4091.259	2197.559	830.599
		ND4	7	3383.273	2033.193	768.475
		ND5	8	6146.210	3146.027	1112.289
		CO3	8	2399.090	1239.251	438.142
	Bipolar Disorder	Total Minor Arc	9	3716.932	2546.736	848.912

		ND4	9	3036.904	2099.002	699.667
		ND5	8	5345.481	3380.925	1195.338
		CO3	8	2233.316	1425.089	503.845
	Schizophrenia	Total Minor Arc	10	4212.717	1973.706	624.141
		ND4	10	3215.950	1596.870	504.975
		ND5	9	6594.850	2835.960	945.320
		CO3	9	2787.915	1222.687	407.562

Supplementary Table 2. Information on sample size, mean, standard deviation, and standard error of the mean for mtDNA content (Figure 2A).

		Brain Regions									
		BA24		CE		HYP		PFC		All Regions	
Diagnosis	Genes	r	p-value	r	p-value	r	p-value	r	p-value	r	p-value
Control	ND4	0.046	0.900	0.092	0.829	0.040	0.888	0.887	0.113	0.130	0.442
	ND5	0.072	0.844	0.241	0.565	0.025	0.930	0.850	0.150	0.226	0.178
	All Genes	0.059	0.805	0.150	0.580	0.024	0.899	0.700	0.053	0.172	0.142
Bipolar Disorder	ND4	-0.150	0.701	-0.451	0.223	0.642	0.033	0.694	0.194	0.405	0.017
	ND5	-0.163	0.675	-0.549	0.100	0.585	0.059	0.581	0.304	0.364	0.032
	All Genes	-0.150	0.551	-0.465	0.045	0.482	0.023	0.578	0.080	0.349	0.003
Schizophrenia	ND4	0.239	0.506	-0.068	0.862	-0.467	0.173	0.341	0.454	0.435	0.008
	ND5	0.299	0.402	-0.237	0.539	-0.300	0.432	0.363	0.480	0.588	0.000
	All Genes	0.206	0.384	-0.139	0.581	-0.380	0.109	0.223	0.464	0.452	0.000
Combined Diagnosis groups	ND4	0.083	0.668	-0.125	0.543	0.133	0.439	0.227	0.390	0.256	0.008
	ND5	0.109	0.574	-0.186	0.352	0.262	0.128	0.325	0.238	0.332	0.001
	All Genes	0.094	0.482	-0.148	0.291	0.161	0.181	0.225	0.223	0.267	0.000

Supplementary Table 3. Complete results for correlation of mtDNA content to NDUFS7 protein expression level in the BA24, CE, HYP, and PFC, separated by gene, brain region and combined. Correlation was evaluated via Pearson correlation test. (Figure 2B).

Brain Region	Diagnosis	Gene	N	Mean	SD	SEM
BA24	Control	CO3	10	0.565	0.141	0.045
		ND4	10	0.734	0.143	0.045
		ND5	10	1.372	0.258	0.082
	Bipolar Disorder	CO3	9	0.573	0.070	0.023
		ND4	9	0.773	0.175	0.058
		ND5	9	1.467	0.172	0.057
	Schizophrenia	CO3	10	0.559	0.098	0.031
		ND4	10	0.788	0.131	0.042
		ND5	10	1.474	0.270	0.085
CE	Control	CO3	8	0.552	0.081	0.029
		ND4	8	0.755	0.204	0.072
		ND5	7	1.444	0.067	0.025
	Bipolar Disorder	CO3	9	0.640	0.092	0.031
		ND4	9	0.795	0.180	0.060
		ND5	9	1.589	0.266	0.089
	Schizophrenia	CO3	9	0.595	0.136	0.045
		ND4	9	0.777	0.093	0.031
		ND5	9	1.424	0.347	0.116
HYP	Control	CO3	15	0.579	0.174	0.045
		ND4	15	0.697	0.185	0.048
		ND5	15	1.447	0.354	0.091

	Bipolar Disorder	CO3	12	0.598	0.100	0.029
		ND4	12	0.781	0.085	0.024
		ND5	12	1.500	0.290	0.084
	Schizophrenia	CO3	9	0.586	0.149	0.050
		ND4	10	0.771	0.116	0.037
		ND5	9	1.380	0.309	0.103
PFC	Control	CO3	7	0.610	0.076	0.029
		ND4	7	0.832	0.210	0.079
		ND5	7	1.619	0.248	0.094
	Bipolar Disorder	CO3	8	0.614	0.116	0.041
		ND4	9	0.835	0.148	0.049
		ND5	8	1.494	0.322	0.114
	Schizophrenia	CO3	9	0.621	0.113	0.038
		ND4	10	0.746	0.181	0.057
		ND5	9	1.471	0.260	0.087

Supplementary Table 4. Complete information on sample size, mean, standard deviation, and standard error of the mean for mtDNA deletion (Figure 3A).

Brain Region	Diagnosis	Gene	N	Mean	SD	SEM
BA24	Control	ND4	9	4.872	1.605	0.535
		ND5	9	4.470	1.863	0.621
		CO3	9	4.343	1.503	0.501
		tRNA	8	1.820	0.360	0.127
		ND1	7	2.258	0.204	0.077
	Bipolar Disorder	ND4	3	4.008	1.104	0.637
		ND5	8	2.123	1.875	0.663
		CO3	8	2.320	1.510	0.534
		tRNA	9	1.983	0.646	0.215
		ND1	9	1.962	0.670	0.223
	Schizophrenia	ND4	9	3.655	1.843	0.614
		ND5	9	3.005	2.014	0.671
		CO3	9	3.326	1.690	0.563
		tRNA	8	2.242	0.747	0.264
		ND1	8	1.999	0.391	0.138
CE	Control	ND4	8	4.686	2.118	0.749
		ND5	8	4.320	2.499	0.884
		CO3	8	4.250	1.683	0.595
		tRNA	8	1.908	0.439	0.155
		ND1	8	1.853	0.324	0.115
	Bipolar Disorder	ND4	7	4.176	2.125	0.803
		ND5	9	3.601	2.291	0.764
		CO3	9	3.541	1.355	0.452

		tRNA	9	1.690	0.320	0.107
		ND1	9	1.860	0.267	0.089
	Schizophrenia	ND4	5	2.929	1.803	0.806
		ND5	6	1.015	0.773	0.316
		CO3	7	2.212	1.376	0.520
		tRNA	8	1.833	0.560	0.198
		ND1	8	2.064	0.548	0.194
HYP	Control	ND4	11	4.763	1.408	0.424
		ND5	13	4.318	2.701	0.749
		CO3	13	3.668	1.777	0.493
		tRNA	14	1.772	0.694	0.185
		ND1	14	1.850	0.583	0.156
	Bipolar Disorder	ND4	10	3.929	1.405	0.444
		ND5	10	3.285	1.952	0.617
		CO3	10	3.327	1.512	0.478
		tRNA	11	2.106	0.665	0.201
		ND1	11	1.936	0.295	0.089
	Schizophrenia	ND4	8	4.949	2.200	0.778
		ND5	9	3.478	2.734	0.911
		CO3	9	3.713	2.050	0.683
		tRNA	9	2.015	0.495	0.165
		ND1	10	2.023	0.411	0.130
PFC	Control	ND4	7	4.425	2.765	1.045
		ND5	6	4.176	2.428	0.991

		CO3	5	3.307	1.403	0.627
		tRNA	8	2.155	1.436	0.508
		ND1	8	1.856	0.421	0.149
	Bipolar Disorder	ND4	6	4.918	2.067	0.844
		ND5	8	4.004	2.211	0.782
		CO3	8	3.793	1.900	0.672
		tRNA	9	2.120	1.063	0.354
		ND1	9	1.691	0.644	0.215
	Schizophrenia	ND4	7	4.023	1.004	0.379
		ND5	8	2.835	1.995	0.705
		CO3	8	3.272	1.709	0.604
		tRNA	10	1.987	1.083	0.342
		ND1	10	1.843	0.421	0.133

Supplementary Table 5. Complete information on sample size, mean, standard deviation, and standard error of the mean for mtDNA oxidation (Figure 3B).
